# RotaC: A web-based tool for the complete genome classification of group A rotaviruses

**DOI:** 10.1186/1471-2180-9-238

**Published:** 2009-11-23

**Authors:** Piet Maes, Jelle Matthijnssens, Mustafizur Rahman, Marc Van Ranst

**Affiliations:** 1 Clinical Virology, Rega Institute for Medical Research, Katholieke Universiteit Leuven, Minderbroedersstraat 10, B3000 Leuven, Belgium

## Abstract

**Background:**

Group A rotaviruses are the most common cause of severe diarrhea in infants and children worldwide and continue to have a major global impact on childhood morbidity and mortality. In recent years, considerable research efforts have been devoted to the development of two new live, orally administered vaccines. Although both vaccines have proven to confer a good protection against severe rotavirus gastroenteritis, these vaccines will have to be screened and may have to be updated regularly to reflect temporal and spatial genotype fluctuations. In this matter, the genetic characterization of circulating and new emerging rotavirus strains will need to be compulsory and accurate. An extended classification system for rotaviruses in which all the 11 genomic RNA segments are used, has been proposed recently. The use of this classification system will help to elucidate the role of gene reassortments in the generation of genetic diversity, host range restriction, co-segregation of certain gene segments, and in adaptation to a new host species.

**Results:**

Here we present a web-based tool that can be used for fast rotavirus genotype differentiation of all 11 group A rotavirus gene segments according to the new guidelines proposed by the *Rotavirus Classification Working Group *(RCWG).

**Conclusion:**

With the increasing sequencing efforts that are being conducted around the world to unravel complete rotavirus genomes of human and animal origin, this tool will be of great help to analyze and correctly classify the large amount of new data. The web-based tool is freely available at http://rotac.regatools.be.

## Background

Group A rotaviruses are the major etiological agent of severe diarrhea in infants and young children worldwide, leading to significant morbidity and mortality. More than 125 million infants and young children develop rotavirus diarrhea globally each year, resulting in 440.000 deaths in children, mostly in the developing countries [1]. Although the infant mortality rate due to rotavirus disease is low in developed countries, the consequences of the disease can be very costly and cause a significant economic burden, which can be both direct (medical costs, outpatient visits, diagnosis, medication) and indirect (lost working hours of parents). For example, the costs associated with rotavirus diarrhea in the United States were estimated at $100-400 million to the healthcare system and $1 billion to the society [2,3].

Extensive genetic variation and reassortment of the 11 double-stranded RNA rotaviral genome segments has resulted in the presence of a large spectrum of different rotavirus genotypes in humans and animals. Rotaviruses, which form a separate genus in the family *Reoviridae*, are divided into seven (A to G) antigenically distinct groups that infect mammalian and avian species, of which group A rotaviruses are the most important due to their high prevalence and pathogenicity in both mammalian and avian species. Group A rotaviruses have a genome that is enclosed in a triple-layered icosahedral capsid, consisting of 11 segments encoding six viral structural proteins (VP1 to VP4, VP6, and VP7) and six nonstructural proteins (NSP1 to NSP6) [4]. Like influenza viruses, a dual classification system for group A rotaviruses has been established depending on two outer capsid proteins VP4 and VP7, defining respectively P en G genotypes. Recently, a genotyping system based on complete nucleotide sequences of all 11 genomic RNA segments has been proposed by Matthijnssens and colleagues [5]. In this new classification system, nucleotide identity cut-off percentages were defined to identify different genotypes for each of the 11 segments (Table 1). Likewise, a nomenclature for the comparison of complete rotavirus genomes was considered in which the notation Gx-P [x]-Ix-Rx-Cx-Mx-Ax-Nx-Tx-Ex-Hx (with x indicating the number of the genotype) is used for the VP7, VP4, VP6, VP1, VP2, VP3, NSP1, NSP2, NSP3, NSP4, and NSP5 encoding genes, respectively. In this new group A rotavirus classification system, the complete open reading frame (ORF) of a rotavirus gene is compared to other complete ORFs of cognate genes available in the GenBank database. If pairwise nucleotide identities between the gene of the novel strain under investigation (strain A) and the strains belonging to an established genotype X are above the cut off value of that gene segment (Table 1), strain A can be assigned to genotype X. The exact relationship between the gene of strain A and cognate genes of all established genotypes, has to be obtained phylogenetically. When all the pairwise nucleotide identities between a gene of the new strain B, and the cognate genes of all the established genotypes are below the cut-off value for that gene segment (Table 1), strain B may be the prototype of a new genotype [6]. If only a partial ORF sequence of a rotavirus genome segment is available, assigning it to a specific genotype is less certain because the genotypic diversity across the ORF is not a constant value. Some regions of the ORF may be highly variable, while others may be more conserved. Since the cut-off percentage values for each of the 11 genome segments has been calculated based on entire ORFs, applying these cut-off percentages to only a part of the ORF, might lead to erroneous conclusions. In accordance with the recommendations of the RCWG, only under certain circumstances when all three of the following restrictions are obeyed, a partial gene sequence might be used to assign a rotavirus gene to an established genotype: (a) at least 50% of the ORF sequence should be determined; (b) at least 500 nucleotides of the ORF should be determined; and (c) identity between strain X and a strain belonging to an established genotype A should be at least 2% above the appropriate cut-off sequence (Table 1), before strain X can be assigned to genotype A.

**Table 1:** Nucleotide identity percentage cutoff values defining genotypes for 11 rotavirus gene segments [5].

Gene product	Cutoff value (%)	Genotype (n)^a^	Description of gene product
**VP7**	80	G (24)	Glycolylated
**VP4**	80	P (33)	Protease sensitive
**VP6**	85	I (14)	Intermediate capsid shell
**VP1**	83	R (6)	RNA-dependent RNA polymerase
**VP2**	84	C (6)	Core shell protein
**VP3**	81	M (7)	Methyltransferase
**NSP1**	79	A (16)	Interferon antagonist
**NSP2**	85	N (6)	NTPase
**NSP3**	85	T (8)	Translation enhancer
**NSP4**	85	E (12)	Enterotoxin
**NSP5**	91	H (8)	Phosphoprotein

^a ^current number of genotypes, which will be updated when new genotypes are assigned by the RCWG.

## Implementation

The classification tool for group A rotaviruses (RotaC v1.0) is written in java with a simple object model in order to make it easy to maintain the code. The interface of the website is written in perl. The RotaC tool can analyze up to a 1000 nucleotide sequences in 'strict' FASTA-format (a first line with a sequence identifier preceded by '>', followed by a second line with the sequence). The analysis of nucleotide sequences with a length below 500 bases is not suitable according to the RCWG guidelines and is not allowed in the RotaC tool.

The genotyping process consists of several subsequent steps. In a first step, the appropriate gene segment is identified by comparing the query sequence with a full genome reference alignment consisting of well-characterized group A rotavirus sequences and by the neighbor-joining algorithm. After the recognition of the segment of origin, the query sequence is aligned using the profile alignment functions of Clustal W v2.0[7] with a reference alignment of the appropriate segment (detailed information about the alignments used with the RotaC tool can be found on http://rotac.regatools.be). In a second step, a distance matrix, based on pairwise alignments with the Needleman-Wunsch algorithm [8], and a phylogenetic tree based on the neighbor-joining algorithm using the Paup* software [9] are constructed and analyzed to identify the genotype of the query sequence by using the nucleotide identity cut-off values summarized in Table 1. The reliability of the clustering of the neighbor-joining tree is assessed using 100 bootstrap replicates, considering 70% as the cut-off value. If the query sequence has a shared identity of at least 3% above the appropriate cut-off value with an established genotype, the query sequence is considered as a member of that specific genotype. If the shared identity is at least 3% below the cut-off value, the query sequence is considered as a new genotype of the proper rotavirus segment. For identities less than 3% below or above the cut-off value, the tool provides only tentative conclusions. In this case, it is recommended to send the sequence to the Rotavirus Classification Working Group for further phylogenetic analysis and correct identification of the genotype. For queries covering less than 50% of the ORF region, no conclusion will be drawn. The user should pay attention that the assignment of a genotype to the query sequence with identities less than 3% below or above the genotype cut-off values should be confirmed by more extensive phylogenetic analysis, or should be send to the RCWG for further analyses and/or validation.

## Results and Discussion

Due to the very limited number of completely sequenced rotavirus genomes, studies on reassortments have been limited to a few gene segments. Recently, the increased availability of complete rotavirus genome sequences, and the introduction of an extended classification and nomenclature system, comprising all 11 rotavirus gene segments, has prompted many investigators to start complete rotavirus genome sequencing projects. Both reassortments between strains belonging to the same host species, and between strains belonging to different host species have been documented several times in the past [10-12]. The new classification system creates a necessary framework to thoroughly analyze possible interspecies transmissions of whole rotaviruses from one host to another, and to study the effect of reassortments on the generation of genetic rotavirus diversity, host range restriction, co-segregation of certain gene segments, and adaptation to a new host species [5]. A *Rotavirus Classification Work Group *was setup to evaluate potentially new genotypes that will be discovered when more and more complete rotavirus genomes from multiple host species will be sequenced [6]. The analyses of complete rotavirus genomes, and the assignment to the appropriate genotypes will be highly facilitated by the use of the free online RotaC-tool. The RotaC-tool will be updated regularly, an will work closely together with the RCWG in order to update the tool with new genotypes, to reflect all established and new genotypes.

## Conclusion

There are several useful web-based tools and database resources for the genotyping analysis of viral sequences, based on phylogenetic trees, or sequence similarities of whole/partial sequences for the genotyping of HIV-1/HIV-2, HTLV-1/HTLV-2, hepatitis B virus, hepatitis C virus and poliovirus sequences [13-17]. Here we have introduced a reliable and easy-to-use automated classification tool for group A rotaviruses. Our RotaC classification tool is in agreement with the rotavirus classification strategy and guidelines as proposed by the Rotavirus Classification Working Group. The web-based RotaC tool can be freely accessed at http://rotac.regatools.be.

## Availability and requirements

• **Project name**: RotaC, Rotavirus Classification Tool v1.0

• **Project home page**: http://rotac.regatools.be

• **Operating system**: platform independent

• **Programming language**: java, perl and PHP

• **License**: none

• **Restrictions to use by non-academics**: none

## Authors' contributions

PM conceived the study, designed the analytical procedure and wrote the software. The paper was written by PM, JM, and MR. All authors read and approved the final manuscript.
